# Spectrum of *PRSS1*, *SPINK1*, *CTRC*, *CFTR*, and *CPA1* Gene Variants in Chronic Pancreatitis Patients in Russia

**DOI:** 10.17691/stm2023.15.2.06

**Published:** 2023-03-29

**Authors:** M.M. Litvinova, K.F. Khafizov, A.S. Speranskaya, A.D. Matsvay, A.Yu. Asanov, K.A. Nikolskaya, L.V. Vinokurova, E.A. Dubtsova, M.G. Ipatova, T.F. Mukhina, M.A. Karnaushkina, D.S. Bordin

**Affiliations:** Associate Professor, Deputy Head for Research Work, Department of Medical Genetics1; Clinical Geneticist; The Loginov Moscow Clinical Scientific Center of Moscow Healthcare Department, 86 Entuziastov Shosse, Moscow, 111123, Russia;; Head of the Laboratory of Genomic Research; Central Research Institute of Epidemiology, Russian Federal Service for Surveillance on Consumer Rights Protection and Human Wellbeing, 3A Novogireevskaya St., Moscow, 111123, Russia;; Senior Researcher, Head of the Laboratory of Multiomix Investigations; Scientific Research Institute for Systems Biology and Medicine, Russian Federal Service for Surveillance on Consumer Rights Protection and Human Wellbeing, 18 Nauchniy Proezd, Moscow, 117246, Russia; Researcher; Lomonosov Moscow State University, 1 Leninskiye Gory, Moscow, 119991, Russia;; Researcher, Laboratory of Genomic Methods Development; Centre for Strategic Planning and Management of Biomedical Health Risks of FMBA of Russia,; Professor, Department of Medical Genetics; I.M. Sechenov First Moscow State Medical University (Sechenov University), 8/2 Malaya Trubetskaya St., Moscow, 119991, Russia;; Physician; The Loginov Moscow Clinical Scientific Center of Moscow Healthcare Department, 86 Entuziastov Shosse, Moscow, 111123, Russia;; Gastroenterologist; The Loginov Moscow Clinical Scientific Center of Moscow Healthcare Department, 86 Entuziastov Shosse, Moscow, 111123, Russia;; Head of the Department of Pathology of the Pancreas and Bile Ducts; The Loginov Moscow Clinical Scientific Center of Moscow Healthcare Department, 86 Entuziastov Shosse, Moscow, 111123, Russia;; Associate Professor, Department of Hospital Pediatrics named after Academician V.A. Tabolin; Pirogov Russian National Research Medical University, 1 Ostrovitianova St., Moscow, 117997, Russia; Head of the Center for the Treatment of Developmental Anomalies and Diseases of the Hepatobiliary System in Children; N.F. Filatov Children’s City Hospital of Moscow Healthcare Department, 15 Sadovaya-Kudrinskaya St., Moscow, 123001, Russia;; Gastroenterologist; Morozovskaya Children’s City Clinical Hospital of Moscow Healthcare Department, 1/9, 4^th^ Dobrininskiy Pereulok, 119049, Moscow, Russia;; Professor, Department of Internal Medicine with a Course of Cardiology and Functional Diagnostics named after Academician V.S. Moiseev; Peoples’ Friendship University of Russia (RUDN University), 6 Miklukho-Maklaya St., Moscow, 117198, Russia;; Professor, Head of the Department of Pancreatic, Biliary and Upper Digestive Tract Disorders; The Loginov Moscow Clinical Scientific Center of Moscow Healthcare Department, 86 Entuziastov Shosse, Moscow, 111123, Russia; Professor, Department of Propaedeutic of Internal Diseases and Gastroenterology; A.I. Yevdokimov Moscow State University of Medicine and Dentistry, 20/1 Delegatskaya St., Moscow, 127473, Russia; Professor, Department of General Medical Practice and Family Medicine; Tver State Medical University, 4 Sovetaskaya St., Tver, 170100, Russia

**Keywords:** chronic pancreatitis, mutations, gene variants, *PRSS1*, *SPINK1*, *CTRC*, *CFTR*, *CPA1*, genetic risk factors, hereditary pancreatitis, idiopathic pancreatitis, Russian population

## Abstract

**The aim of the study** was to define the spectrum of genetic risk factors of chronic pancreatitis (CP) development in patients living in the European part of the Russian Federation.

**Materials and Methods:**

The study group included 105 patients with CP, with the age of the disease onset under 40 years old (the average age of onset was 26.9 years). The control group consisted of 76 persons without clinical signs of pancreatitis. The diagnosis of chronic pancreatitis in patients was made on the basis of clinical manifestations and the results of laboratory and instrumental investigations. Genetic examination of patients was conducted using the next-generation sequencing (NGS) technology and included targeted sequencing of all exons and exon-intron boundaries of the *PRSS1*, *SPINK1*, *CTRC*, *CFTR*, and *CPA1* genes. The genotyping of the rs61734659 locus of the *PRSS2* gene was also conducted.

**Results:**

Genetic risk factors of the CP development were found in 61% of patients. Pathogenic and likely-pathogenic variants associated with the risk of CP development were identified in the following genes: *CTRC* (37.1% of patients), *CFTR* (18.1%), *SPINK1* (8.6%), *PRSS1* (8.6%), and *CPA1* (6.7%). The frequent gene variants in Russian patients with CP were as follows: *CTRC* gene — c.180C>T (rs497078), c.760C>T (rs121909293), c.738_761del24 (rs746224507); cumulative odds ratio (OR) for all risk alleles was 1.848 (95% CI: 1.054–3.243); *CFTR* gene — c.3485G>T (rs1800120), c.1521_1523delCTT (p.Phe508del, rs113993960), and c.650A>G (rs121909046); OR=2.432 (95% CI: 1.066–5.553). In the *SPINK1*, *PRSS1*, and *CPA1* genes, pathogenic variants were found only in the group of patients with CP. The frequent variants of the *SPINK1* gene include c.101A>G (p.Asn34Ser, rs17107315) and c.194+2T>C (rs148954387); of the *PRSS1* gene — c.86A>T (p.Asn29Ile, rs111033566); of the *CPA1* gene — c.586-30C>T (rs782335525) and c.696+23_696+24delGG. The OR for the CP development for the c.180TT genotype (rs497078) *CTRC* according to the recessive model (TT vs. CT+CC) was 7.05 (95% CI: 0.86–263, p=0.011). In the *CTRC* gene, the variant c.493+49G>C (rs6679763) appeared to be benign, the c.493+51C>A (rs10803384) variant was frequently detected among both the diseased and healthy persons and did not demonstrate a protective effect. The protective factor c.571G>A (p.Gly191Arg, rs61734659) of the *PRSS2* gene was detected only in the group of healthy individuals and confirmed its protective role. 12.4% of the patients with CP had risk factors in 2 or 3 genes.

**Conclusion:**

Sequencing of the coding regions of the *PRSS1*, *SPINK1*, *CTRC*, *CFTR*, and *CPA1* genes allowed to identify genetic risk factors of the CP development in 61% of cases. Determining the genetic cause of CP helps to predict the disease course, perform preventive measures in the proband’s relatives, and facilitate a personalized treatment of the patient in future.

## Introduction

Chronic pancreatitis (CP) is characterized by a chronic inflammatory process in the pancreatic tissue, which, with the disease progression, is replaced by fibrous tissue. The incidence of CP in adults is 1 per 2632 persons, in children — 1 per 7692 [1, 2]. However, due to various reasons, there may be a certain part of unrecognized cases of this disease.

In general, risk factors contributing to the CP development are well known. They include smoking, excessive alcohol drinking, cholelithiasis, exposure to stress factors, hypertriglyceridemia, hypercalcemia, injuries and malformations of the pancreas, taking of certain medications, etc. [3]. However, in clinical practice, there are often situations when it is not possible to specify the exact cause of the disease. In such cases, “idiopathic pancreatitis” is diagnosed. For these patients, conduction of a genetic examination is of utmost importance, as it is possible to identify a hereditary form of CP in approximately 20% of cases [2, 4].

According to the latest version of the classification of the main CP causes (TIGAR-O Version 2), genetic factors play a significant role in the disease pathogenesis [5].

Products of the following genes: *PRSS1* (cationic trypsinogen type 1), *SPINK1* (secretory pancreatic inhibitor of trypsin type 1), *CTRC* (chymotrypsin C), *CFTR* (cystic fibrosis transmembrane conductance regulator), and *CPA1* (carboxypeptidase A1) are essential for the pancreas functioning [4].

**The aim of the study** was to determine the structure of chronic pancreatitis genetic causes in patients living in the European part of Russia by sequencing the entire coding sequence of the *PRSS1*, *SPINK1*, *CTRC*, *CFTR*, and *CPA1* genes.

## Materials and Methods

The study group included 105 patients with CP with the age of disease onset under 40 years old (the average age at the time of blood sampling was 35.1 years; the average age of CP manifestation was 26.9 years). Of the patients, 65 were males (average age at the time of blood sampling was 36.3 years; average age of CP onset was 28.7 years) and 40 were females (average age at the time of blood sampling was 33.2 years; average age of CP onset was 23.9 years). The control group included 76 persons of the corresponding gender and age without pancreatitis (average age was 31.6 years).

All study participants provided their voluntary informed consent to participate in the study. The work was approved by the local ethics committee of the Loginov Moscow Clinical Scientific Center of Moscow Healthcare Department (Russia) (protocol No.8 of 2015) and complies with the requirements of the Declaration of Helsinki (2013).

The diagnosis of chronic pancreatitis was made on the basis of clinical manifestations and the results of laboratory and instrument investigations. The genetic study was conducted using the next-generation sequencing (NGS) technology, which included targeted sequencing of all exons and exon-intron bounderies of the *PRSS1* (5 exons), *SPINK1* (4 exons), *CTRC* (8 exons), *CFTR* (27 exons), and *CPA1* (10 exons) genes. Moreover, in this study we decided to investigate the role of the protective factor rs61734659 of the *PRSS2* gene in relation to the CP development in Russian patients, that is why this gene region was included in the developed targeted genetic panel.

For genetic analysis, DNA was isolated from venous whole blood samples of the study participants using the DNeasy Blood & Tissue Kit (QIAGEN, Germany) on an automated system for the DNA, RNA, and proteins isolation QIAcube (QIAGEN, Germany) according to the manufacturer’s standard protocol.

Sample preparation for sequencing included a PCR test with a primer panel of 67 primer pairs (with a total coverage of 22,693 nucleotides), which was developed specifically for this study with the Ion AmpliSeq Designer software (Thermo Fisher Scientific, USA). The nucleotide sequences of the analyzed genes were received from the NCBI database (https://www.ncbi.nlm.nih.gov/). The targeted amplification was conducted using reagents for PCR tests (AmpliSense; Central Research Institute of Epidemiology of the Russian Federal Service for Surveillance on Consumer Rights Protection and Human Wellbeing, Russia) on a QuantStudio 5 Real-Time PCR Systems machine (Thermo Fisher Scientific, USA).

The NGS libraries were prepared using T4 Polynucleotide Kinase (New England Biolabs, USA) and T4 DNA Ligase (New England Biolabs, USA) reagents according to the manufacturer’s instructions with minor modifications.

At all stages, purification of the PCR product and NGS libraries from components of the reaction mixtures was conducted using carboxylated magnetic particles Sera-Mag SpeedBeads (Sigma-Aldrich, USA). The DNA concentration of the isolated samples, the PCR product, and the final libraries was measured fluorometrically using the Qubit dsDNA HS Assay Kit (Thermo Fisher Scientific, USA) on a Qubit 2.0 Fluorometer (Thermo Fisher Scientific, USA). The quality of the final libraries was assessed on a chip by capillary electrophoresis on Agilent 2100 Bioanalyzer (Agilent Technologies, USA) using an Agilent High Sensitivity DNA Kit (Agilent Technologies, USA).

Sequencing on the Ion S5 platform (Thermo Fisher Scientific, USA) was conducted using an Ion 520 & Ion 530 Kit-Chef (Thermo Fisher Scientific, USA) and a semiconductor chip Ion 530™ Chip Kit (Thermo Fisher Scientific, USA).

Bioinformatic processing of the NGS sequencing data included deletion of low-quality reads using the PRINSEQ-lite software [6]; mapping to the reference human genome (GRCh38.p7, PRJNA31257) with Burrows–Wheeler Aligner (BWA-MEM, v_0.7.13) [6]; search for nucleotide sequence variants using the Genome Analysis Toolkit (GATK version 4.0.11.0) software package [7]. SAMtools v_1.3.1 [8] and Picard toolkit v_2.18.17 were used to handle sam/bam files; the VEP software [9] using the 94_GRCh38 cache was applied for primary annotation of variants.

Validation of nucleotide sequence variants detected by automatic analysis was conducted manually using the Tablet graphical user interface for viewing assemblies and alignments [10].

Clinical interpretation of the identified variants was conducted using the following electronic resources and databases: dbSNP (https://www.ncbi.nlm.nih.gov/snp/), ClinVar (https://www.ncbi.nlm.nih.gov/clinvar/), Cystic Fibrosis Mutation Database (http://www.genet.sickkids.on.ca), CFTR2 (https://cftr2.org), CFTR-France (https:// cftr.iurc.montp.inserm.fr), OMIM (https://www.omim.org/), pancreasgenetics.org.

**Statistical analysis** was conducted using the standard R software package [11]. To specify the association between polymorphisms of the analyzed genes and the CP development, the odds ratio (OR) and the relative risk (RR) were calculated using the standard formulas with the calculation of a 95% confidence interval (CI). To determine the significance level of value differences, Student’s t-test was applied with the calculation of χ^2^ and the p-value of significance. Differences between the samples were considered statistically significant at p≤0.05. The standard deviation of the percentage was calculated by the formula:

σp=p(1−p)n,

where *p* is the percentage, *σ_p_* is the standard deviation of the percentage, and *n* is the number of assessments.

## Results

### Variants of the CTRC gene

Most often, genetic risk factors of the disease development were identified in the *CTRC* gene. In the majority of cases, patients were heterozygous (n=22) or homozygous (n=8) carriers of the so-called risk diplotype c.180C>T (rs497078)/ c.493+52G>A (rs545634), which was found in 30 patients. In three more patients with CP, the c.180C>T (rs497078) variant was identified without combination with c.493+52G>A (rs545634): in two cases in the heterozygous and in one case in the homozygous state. Thus, 33 persons out of 105 (31.4%) had the c.180C>T (rs497078) variant of the *CTRC* gene. The second frequent finding in the *CTRC* gene in patients with CP was a gene variant of unknown clinical significance — c.493+49G>C (rs6679763), which was identified in five persons in the heterozygous state (4.8%). Four other patients (3.8%) had pathogenic variant c.760C>T (p.Arg254Trp, rs121909293) in the heterozygous state, and two patients (1.9%) had pathogenic variant c.738_761del24 (p.Lys247_Arg254del, rs746224507) in heterozygous state as well. One patient had a gene variant of unknown clinical significance — c.-59C>T (rs183658182) in the heterozygous form (Table 1).

**Table 1:** Frequency of alleles and genotypes of polymorphic loci of the *CTRC* gene in patients with chronic pancreatitis and healthy individuals

Variant	Genotype/allele	Patients with chronic pancreatitis (n=105)	Control group (n=76)	p
c.180C>T (rs497078)/ c.493+52G>A (rs545634)	CT/GA TT/AA CT/GG TT/GG CT/GA+CT/GG TT/AA+TT/GG CT/GA+CT/GG+TT/AA+TT/GG	22 8 2 1 24 9 33	16 0 4 0 20 0 20	0.987 0.014 0.213 0.394 0.593 0.009 0.456
	c.180C c.180T	168 42	132 20	0.089 0.089
с.493+49G>C (rs6679763)	GG GC CC	100 5 0	68 8 0	0.139 0.139 1
	G C	205 5	144 8	0.146 0.146
c.760C>T (p.Arg254Trp) rs121909293	CC CT TT	101 4 0	75 1 0	0.313 0.313 1
	C T	206 4	151 1	0.316 0.316
c.738_761del24 (p.Lys247_Arg254del, rs746224507)	NN NDel DelDel	103 2 0	76 0 0	0.227 0.227 1
	N Del	208 2	152 0	0.228 0.228
c.-59C>T (rs183658182)	CC CT TT	104 1 0	75 1 0	0.818 0.818 1
	C T	209 1	151 1	0.818 0.818
c.493+51C>A (rs10803384)	CC CA AA	86 18 1	60 15 1	0.620 0.656 0.818
	C A	190 20	135 17	0.607 0.607

In the control group, 21 individuals (27.6%) had changes in the *CTRC* gene in the heterozygous state. Of them, 16 persons were heterozygous carriers of the c.180C>T (rs497078) gene variant in the form of the diplotype c.180C>T (rs497078)/c.493+52G>A (rs545634) variant, four persons were heterozygous carriers of the c.180C>T variant (rs497078) without c.493+52G>A (rs545634) variant, one person had the c.760C>T (p.Arg254Trp, rs121909293) gene variant (see Table 1).

Comparison of the frequency of c.180T allele (rs497078) in the group of patients with CP (20%) and the control group (13.2%) showed that differences were statistically insignificant (χ^2^=2.908; p=0.089). However, homozygosity for the c.180C>T (rs497078)/ c.493+52G>A (rs545634) diplotype was observed only in patients with CP (see Table 1). This indicates a significant effect of the homozygous genotype for the c.180C>T variant (rs497078) of the *CTRC* gene on the development of inflammatory changes in the pancreas. The OR for the c.180TT genotype according to the recessive model (TT vs. CT+CC) was 7.05 (95% CI: 0.86–263, p=0.011).

It is of interest to note that the c.493+49G>C variant (rs6679763) was identified in a heterozygous form in 8 out of 76 healthy persons (10.5%). When comparing the frequencies of different genotypes and alleles for the rs6679763 polymorphic locus, no statistically significant differences were detected between the group of patients and the control group (p=0.139 and p=0.146, respectively). This fact leads to a conclusion that this variant has no pathogenic effect on the risk of the CP development.

The variant of unknown clinical significance c.-59C>T (rs183658182) in the *CTRC* gene was identified in a heterozygous form in one person from the control group, which can be a sign of its low significance for the CP development. The c.760C>T variant (p.Arg254Trp, rs121909293) was found in one person from the control group (a 23-year-old woman without manifestations of CP at the time of blood sampling). The c.738_761del24 variant (p.Lys247_Arg254del, rs746224507/rs515726210) was not detected in individuals from the control group.

In addition to the above-mentioned findings, both in the group of patients with CP and in the control group we found the *CTRC* gene frequent polymorphism c.493+51C>A (rs10803384), which, according to the information from the pancreasgenetics.org database, has a protective role in the disease development. In this study, no significant differences in the frequency of genotypes and alleles for this *CTRC* polymorphic locus were identified between the two groups (see Table 1).

To summarize, pathogenic variants c.760C>T (p.Arg254Trp, rs121909293) and c.738_761del24 (p.Lys247_Arg254del, rs746224507) and genetic risk factors c.180C>T (rs497078)/c.493+52G>A (rs545634) of the *CTRC* gene were detected in 39 out of 105 examined patients with CP (37.1%).

The most frequent and significant variants of the *CTRC* gene identified in patients with CP were as follows: the c.180C>T (rs497078)/c.493+52G>A (rs545634) diplotype and the c.180C>T (rs497078) variant — 84.6% of all findings in this gene; c.760C>T (p.Arg254Trp, rs121909293) — 10.3% of all findings in this gene; c.738_761del24 (p.Lys247_Arg254del, rs746224507) — 5.1% of all findings in this gene.

The frequency of detection of any of the above-mentioned risk alleles of the *CTRC* gene in the group of patients with CP was 22.9% (48 alleles out of 210), whereas the same parameter for the control group was 13.8% (21 alleles out of 152) (χ^2^=4.672; p=0.031). The OR for CP development in case of the risk allele of the *CTRC* gene was 1.848 (95% CI: 1.054– 3.243; standard error of the mean — 0.287).

### Variants of the CFTR gene

19 out of 105 patients with CP (18.1%) had pathogenic or likely-pathogenic variants of the *CFTR* gene in different combinations: c.2620-6T>C, c.224G>A (p.Arg75Gln), c.451C>A (p.Gln151Lys), c.650A>G (p.Glu217Gly), c.1079C>T (p.Thr360Ile), c.2012delT (p.Leu671Ter), c.1516A>G (p.Ile506Val), c.1521_1523delCTT (p.Phe508del), c.1399C>T (p.Leu467Phe), c.1584G>A (p.Glu528=), c.3485G>T (p.Arg1162Leu), c.2991G>C (p.Leu997Phe), c.1969A>G (p.Arg657Gly), c.2619+86delT. Three pathogenic variants, c.3485G>T (p.Arg1162Leu), c.1521_1523delCTT (p.Phe508del), and c.650A>G (p.Glu217Gly), were repetitive and were identified in seven patients (6.7%). One of the *CFTR* gene variants (c.2619+86delT) was detected for the first time in the study in five patients (in all cases, in the homozygous form). More detailed information about the patients and the variants of the *CFTR* gene identified in them can be found in our earlier publication [12].

In the control group, pathogenic or likely-pathogenic variants of the *CFTR* gene were also identified. For instance, 8 out of 76 (10.5%) individuals without symptoms of pancreatitis had variants c.650A>G (p.Glu217Gly), c.3854C>T (p.Ala1285Val), c.443T>C (p.Ile148Thr), c.1584G>A (p.Glu528=), c.224G>A (p.Arg75Gln), c.3532_3535dup (p.Thr1179IlefsTer17) in the heterozygous state.

The frequency of detection of the pathologically altered allele of the *CFTR* gene in the group of patients with CP was 11.9% (25 alleles out of 210), whereas the same parameter for the control group was 5.3% (8 alleles out of 152) (χ^2^=4.695; p=0.031). The OR of the CP development in case of the presence of the pathological allele of the *CFTR* gene in the genotype was 2.432 (95% CI: 1.066–5.553; standard error of the mean — 0.421).

### Variants of the SPINK1 gene

Pathogenic and likely-pathogenic variants of the *SPINK1* gene were identified only in the group of patients with CP (all variants were found in the heterozygous state). Eight patients (7.6%) were carriers of the known pathogenic variants of the *SPINK1* gene: 6 patients had pathogenic variant c.101A>G (p.Asn34Ser, rs17107315) combined with the risk factor c.56-37T>C (rs17107318), 2 patients had variant c.194+2T>C (rs148954387) (Table 2).

**Table 2:** Characteristics of the *SPINK1* gene variants in Russian patients with chronic pancreatitis

Variant*, rs	Patients with chronic pancreatitis (n=105)	Frequency of the alternative allele in the chronic pancreatitis group (210 alleles) (%)	Frequency of the alternative allele in the European population (%)	ClinVar	pancreasgenetics.org
c.101A>G (p.Asn34Ser, rs17107315) combined with the risk factor c.56-37T>C (rs17107318)	6 (het)	2.9	0.980	Conflicting interpretations of pathogenicity: association; risk factor; pathogenic (10); established risk allele (1); uncertain significance (3); likely benign (1)	Pathogenic
c.194+2T>C (rs148954387)	2 (het)	1.0	0.005	Conflicting interpretations of pathogenicity: pathogenic (8); uncertain significance (1)	Pathogenic
c.126A>G (p.Ile42Met, rs370266754)	1 (het)	0.5	0.015	Uncertain significance	Unknown
c.88-23A>T (rs199929811)	1 (het)	0.5	0.30	Benign/likely benign	Benign

* These *SPINK1* gene variants were not found in the control group; het — heterozygous state.

Moreover, a variant of unknown clinical significance c.126A>G (p.Ile42Met, rs370266754) was found in one case, and the c.88-23A>T (rs199929811) variant was identified in two patients. The latter variant, according to the pancreasgenetics.org database, is benign and does not increase the risk of CP development. In our study, this variant (rs199929811) was not identified in the control group, which leads to the need to revise the data on the clinical interpretation of this variant.

Therefore, without the benign variant rs199929811 of the *SPINK1* gene, 9 out of 105 patients with CP (8.6%) were carriers of pathogenic variants. The most frequent variants include: c.101A>G (p.Asn34Ser, rs17107315) combined with the risk factor c.56-37T>C (rs17107318) — 66.7% of all findings in this gene and c.194+2T>C (rs148954387) — 22.2% of all findings in this gene.

### Variants of the PRSS1 gene

Similar to the *SPINK1* gene, pathogenic and likely-pathogenic variants of the *PRSS1* gene were identified only in the group of patients with CP (all of them are in the heterozygous state).

5 out of 105 patients with CP (4.8%) had known pathogenic variants of the *PRSS1* gene: 3 patients were carriers of the c.86A>T (p.Asn29Ile, rs111033566) variant, 1 patient had the c.365G>A (p.Arg122His, rs111033565) variant, and 1 more patient had variant c.68A>G (p.Lys23Arg, rs111033567) (Table 3).

**Table 3:** Characteristics of the *PRSS1* gene variants in Russian patients with chronic pancreatitis

Variant*, rs	Patients with chronic pancreatitis (n=105)	Frequency of the alternative allele in the chronic pancreatitis group (210 alleles) (%)	Frequency of the alternative allele in the European population (%)	ClinVar	pancreasgenetics.org
c.86A>T (p.Asn29Ile, rs111033566)	3 (het)	2.9	18.0	Pathogenic	Pathogenic
c.365G>A (p.Arg122His, rs111033565)	1 (het)	0.5	0.001	Pathogenic	Pathogenic
c.68A>G (p.Lys23Arg, rs111033567)	1 (het)	0.5	—	Likely pathogenic	Pathogenic
c.455-93T>C (rs569672741)	2 (het)	1.0	0.10	Not reported	Not reported
c.200+32C>T (rs182426777)	2 (het)	1.0	0.30	Not reported	Not reported

* These *PRSS1* gene variants were not found in the control group; het — heterozygous state.

Moreover, there were additional findings during the sequencing of the *PRSS1* gene in the group of patients with CP. Variant c.455-93T>C (rs569672741) was identified in the heterozygous form in two patients. According to the information from the dbSNP database, the frequency of the c.455-93C allele in the European population is 0.1%. In the ClinVar database and in the specialized database pancreasgenetics.org, this variant is not annotated. It should be noted that this *PRSS1* gene substitution was not found in this study in the control group.

A similar situation was observed for the *PRSS1* intron variant c.200+32C>T (rs182426777), which was identified in the heterozygous form in two patients with CP. This variant is not described in the mentioned databases and occurs in the European population with a frequency of approximately 0.3%.

Thus, c.86A>T (p.Asn29Ile, rs111033566) was found in three patients and can be called a major pathogenic variant of the *PRSS1* gene in Russian patients with CP.

The cumulative frequency of risk alleles of the *PRSS1* gene in the group of patients with CP was 4.3% (9 alleles); in the control group, it was null (χ^2^=6.680; p=0.01).

### Variants of the CPA1 gene

Sequencing of the entire coding sequence of the *CPA1* gene revealed several variants that require additional studying.

Two out of 105 patients with CP (1.9%) were heterozygous carriers of the c.586-30C>T variant (rs782335525), which is not annotated in the ClinVar and pancreasgenetics.org databases and was not found in the control samples. This variant occurs in the European population with a frequency of 0.015%.

One patient had the *CPA1* c.-90C>T variant (rs377553060) in the heterozygous state, which was also not described in the mentioned databases and was not found in the control group. This variant occurs in the European population with a frequency of 0.03%.

Three unrelated patients with CP (2.8%) had the c.696+23_696+24delGG variant (chr7 130383816 TGG>TG,T) in the homozygous form. Previously this variant was not described in the literature, it is not annotated in genomic databases, and its frequency is not established. It was not registered in the control samples in this study. Segregation genetic analysis in the families of patients was not conducted.

One patient with CP was a heterozygous carrier of the *CPA1* c.983A>G (p.Glu328Gly) missense variant, which, similar to the previous variant, was identified for the first time in this cohort of patients with CP.

In two patients with CP (1.9%), the c.509_510insGG (p.Ala171GlufsTer121) (chr7 130383416 C>CGG) variant was identified in the heterozygous form. This variant of the *CPA1* gene is not annotated in the global genomic databases. It should be noted that pathogenic variants of the missense type with uncertain clinical significance were previously described in this region of the *CPA1* gene. However, in this study, the c.509_510insGG variant was also identified in a heterozygous state in 5 out of 76 healthy persons of the control group (6.6%), which indicates that there is no association between its presence and an increased risk of pancreatitis development (Table 4).

**Table 4:** Characteristics of the *CPA1* gene variants in Russian patients with chronic pancreatitis

Variant, rs	Patients with chronic pancreatitis (n=105)	Individuals of the control group (n=76)	Frequency of the alternative in the chronic allele pancreatitis group (210 alleles) (%)	Frequency of the alternative allele in the European population (%)	ClinVar	pancreasgenetics.org
c.586-30C>T (rs782335525)	2 (het)	0	1.0	0.015	Not reported	Not reported
c.-90C>T (rs377553060)	1 (het)	0	0.5	0.030	Not reported	Not reported
c.509_510insGG (p.Ala171GlufsTer121) (chr7 130383416 C>CGG)	2 (het)	5 (het)	1.0	Not reported	Not reported	Not reported
c.696+23_696+24delGG	3 (hom)	0	2.9	Not reported	Not reported	Not reported
c.983A>G (p.Glu328Gly)	1 (het)	0	0.5	Not reported	Not reported	Not reported

Note: het — heterozygous state, hom — homozygous state.

Thus, the total frequency of potentially risk alleles of the *CPA1* gene in the group of patients with CP was 4.8% (10 alleles); in the control group, it was 0% (χ^2^=7.444; p=0.007).

### Protective factor of the PRSS2 gene

The protective factor c.571G>A (p.Gly191Arg, rs61734659) of the *PRSS2* gene was identified only in the group of healthy individuals, seven of them were heterozygous carriers of this polymorphism. Thus, the frequency of the c.571A allele in the group of patients with CP was 0%, whereas in the control group it was 4.6% (χ^2^=9.862; p=0.002). That means that the protective effect of the c.571A allele at the rs61734659 polymorphic locus was confirmed.

### Summary data on identified genetic risk factors

26.5% of patients with CP had a family history of this disease.

As a result of the genetic testing, in 64 out of 105 patients (61%) with the age of CP onset under 40 years old, significant molesular-genetic findings were identified in different combinations in the *PRSS1, SPINK1, CTRC, CFTR*, and *CPA1* genes.

Summary data on the results of sequencing with the use of the developed targeted genetic panel is shown in Table 5.

**Table 5:** Frequency of identification of genetic risk factors of chronic pancreatitis development with the use of the used targeted genetic panel

Gene	Patients with chronic pancreatitis (n=105), n/%	Control group (n=76), n/%	p
*CTRC*	39/37.1	21/27.6	0.180
*CFTR*	19/18.1	8/10.5	0.159
*SPINK1*	9/8.6	0	0.009
*PRSS1*	9/8.6	0	0.009
*CPA1*	7/6.7	0	0.022

Note: analysis of the entire coding sequence of the *PRSS1*, *SPINK1*, *CTRC*, *CFTR*, and *CPA1* genes was conducted using the next-generation sequencing (NGS) technology.

Most frequently, patients with CP had *CTRC* gene variants (37.1% of patients). Less frequently we saw pathogenic and likely-pathogenic variants of the *CFTR* (18.1%), *SPINK1* (8.6%), *PRSS1* (8.6%), and *CPA1* (6.7%) genes.

The contribution of *PRSS1*, *SPINK1*, *CTRC*, *CFTR*, and *CPA1* gene variants to the development of CP in a cohort of Russian patients is demonstrated in Table 6, which shows that the OR for the CP development for the *CTRC* and *CFTR* gene variants is 1.848 and 2.432, respectively. The maximum risks of developing chronic inflammation in the pancreas are identified for carriers of pathogenic variants in the *PRSS1, SPINK1*, and *CPA1* genes, which is consistent with the currently accepted autosomal dominant theory of inheritance of *PRSS1*-and *SPINK1*-associated CP (OMIM #167800).

**Table 6:** Association of the risk alleles of *PRSS1*, *SPINK1*, *CTRC*, *CFTR*, and *CPA1* genes with chronic pancreatitis development in the Russian cohort of patients

Gene	Identified risk alleles (n/%)		p	OR
	Patients with chronic pancreatitis (210 alleles)	Control group (152 alleles)		
*CTRC*	48/22.9	21/13.8	**0.031**	1.848
*CFTR*	25/11.9	8/5.3	**0.031**	2.432
*SPINK1*	9/4.3	0	**0.01**	Not applicable
*PRSS1*	9/4.3	0	**0.01**	Not applicable
*CPA1*	10/4.8	0	**0.007**	Not applicable

Note: analysis of the entire coding sequence of the *PRSS1*, *SPINK1*, *CTRC*, *CFTR*, and *CPA1* genes was conducted using the next-generation sequencing (NGS) technology.

Some patients with CP had a combination of pathogenic variants and risk factors in several genes at the same time. For instance, 11 patients were carriers of two genetic risk factors, two patients had three genetic risk factors (Table 7).

**Table 7:** Combination of genetic risk factors in patients with chronic pancreatitis

**Amount of genetic risk factors**	**Patients with chronic pancreatitis (n=105)**	**Frequency, *p*±_σ_*p* (%)**	
0	41	39±7.6	
1	51	61±6.1	79.7±5.6
2 and more	13		20.3±11.2

## Discussion

The present study, which included 105 patients with CP, was characterized by a specific predominance of males, which is consistent with the data from the literature, according to which the ratio of men and women among patients with CP is 1.05 [13].

Based on the assumption that hereditary forms of CP manifest at an earlier age, the group of the study participants included patients with an age of the disease onset under 40 years old. There are publications in the worldwide literature where the authors used the same principle to form the group of the patients for the further investigation [14-16]. For instance, Zou et al. [17] showed that the average age of pancreatitis onset in patients with molecular findings in the *SPINK1*, *PRSS1*, *CTRC*, and *CFTR* genes was 29.70±14.84 years, whereas in patients having no mutations in these genes the first clinical signs of the disease appeared at the age of 43.01±15.97 years [17].

The implementation of this approach allowed to identify genetic predictors of the CP development in the majority (61%) of patients, which is generally consistent with the literature. In particular, when sequencing the *SPINK1*, *PRSS1*, *CTRC*, and *CFTR* genes in a large group of patients with CP, Zou et al. [17] identified pathogenic variants in 50.42% of cases. At that, mutations of the mentioned genes were detected by the authors only in 5.94% of cases (OR=16.12; p<0.001) in the control group [17].

The content of the genetic panel developed in this study is influenced by the results of original studies and data from genomic databases, according to which the *PRSS1*, *SPINK1*, *CTRC*, *CFTR*, and *CPA1* genes play a significant role in molecular pathogenesis of the disease [18]. The *PRSS1* gene is responsible for production of cationic trypsinogen type 1, the *SPINK1* gene is a secretory pancreatic inhibitor of trypsin type 1, the *CTRC* gene is chymotrypsin C, the *CFTR* gene is a cystic fibrosis transmembrane conductance regulator, the *CPA1* gene is associated with abnormal clotting of the carboxypeptidase A1 protein and endoplasmic stress of reticulum in pancreatic cells [4]. It should be noted that hereditary forms of CP associated with mutations of the *PRSS1* (MIM# 276000) and *SPINK1* (MIM# 167790) genes are characterized by an autosomal dominant type of inheritance.

The results of the analysis of the entire coding sequence of these genes using NGS to identify major pathogenic variants and genetic risk factors specific to the Russian population are of interest. Until now, patients with CP in the Russian Federation have not been genotyped so comprehensively. Currently, in most cases, the genetic diagnostics of CP in Russia is limited to finding several frequent mutations in the *PRSS1* and *SPINK1* genes. In certain cases, patients undergo sequencing of the coding regions of the *PRSS1* and *SPINK1* genes, which, according to the data of this study, is insufficient and may omit the CP genetic risk factors in patients in the majority of cases.

Moreover, the results of this study allowed to form a list of genetic markers which can be used as screening for the most common genetic risk factors of CP development in Russian patients.

The most frequent molecular finding among patients with CP were *CTRC* gene variants, which were identified in 37.1% of patients and in this population might be considered as risk factors (see Table 5). The OR of pathology development in case of genetic risk factors of this gene was 1.848 (p=0.031). At the same time, different variants of this gene contribute to the risk of the CP development in different manner.

The c.180C>T (rs497078)/c.493+52G>A (rs545634) diplotype of the *CTRC* gene is widely discussed in the literature and is a known genetic risk factor of the CP development. For instance, LaRusch et al. [19] demonstrated an association of the c.180T allele (rs497078) with the risk of CP development in the European population (OR for the CT genotype was 1.36; for the TT genotype — 3.98). This study identified an association of the c.180C>T (rs497078) polymorphism with the increased risk of CP development only in case of homozygosity for this variant (OR=7.05; p=0.011). The lack of a statistically significant association of rs497078 in heterozygous state with an increased probability of pathology development in this study might be due to the characteristics of the cohort, and particularly to the early CP onset in patients, as a later disease onset is typical for individuals with pathogenic variants of this gene.

Other changes in the *CTRC* gene in patients with CP have a greater impact on the risk of the disease development. For instance, the pathogenic variant c.760C>T (p.Arg254Trp, rs121909293) was found in the heterozygous state in four (3.8%) patients with CP and only in one (1.3%) person from the control group. However, this control sample was obtained from a 23-year-old woman who, due to her age, might not yet have CP onset. These findings correlate with the data of foreign researchers, in particular, with the results of study by Rosendahl et al. [20], in which the frequency of this missense variant in a cohort of German patients with idiopathic and hereditary CP was 2.1%, whereas in the control group it was identified only in 0.6% of cases.

A similar situation was observed for the pathogenic variant c.738_761del24 (p.Lys247_Arg254del, rs746224507/ rs515726210), which was identified in the study only in the group of patients with CP.

Therefore, a relatively rare but recurring variants c.760C>T (p.Arg254Trp, rs121909293) and c.738_761del24 (p.Lys247_Arg254del, rs515726210) have the maximum impact on the risk of CP development in the study cohort.

Moreover, we demonstrated that the c.493+49G>C (rs6679763) variant of the *CTRC* gene, which is described as a variant of unknown significance in the specialized database on genetic findings in CP (pancreasgenetics.org), did not increase the risk of the disease development and therefore was a benign variant.

A frequent variant of the *CTRC* gene, c.493+51C>A (rs10803384), described in the pancreasgenetics. org database as protective factor regarding the CP development, did not show a protective effect in the current study.

Another variant of the *CTRC* gene of unknown clinical significance, c.-59C>T (rs183658182), requires further investigation, and this study’s results cannot attribute it to the risk factors.

Findings in the *CFTR* gene in the group of patients with CP were the second in terms of prevalence. 18.1% of patients were carriers of various variants of this gene. Among all variants, the following are recurring in Russian patients with CP: c.3485G>T (p.Arg1162Leu), c.1521_1523delCTT (p.Phe508del), and c.650A>G (p.Glu217Gly). One of the variants of the *CFTR* gene (c.2619+86delT), first discovered in this study in five patients with CP in the homozygous form, requires further investigation in terms of both prevalence and functional significance. Detailed information on *CFTR* gene variants and patients with *CFTR*-related CP can be found in the article published by Litvinova et al. [12]. The cumulative OR of the CP development with a pathological *CFTR* allele was 2.432, which in general is consistent with the study by Zou et al. [17], where the same parameter was 3.71.

Pathogenic changes in the *SPINK1* and *PRSS1* genes were identified in 8.6% of patients with CP. The present data differ from the level of detection of mutations in these two genes shown by Masamune et al. [21], where 41% of patients were carriers of pathogenic variants of the *PRSS1* gene and 36% of patients had mutations in the *SPINK1* gene. Potentially, this difference is due to the fact that the present study did not focus on selection of patients with a family history of pancreatitis, whereas the mentioned study conducted in Japan (231 samples) included patients with a family history of CP.

Pathogenic variants of the *SPINK1* and *PRSS1* genes have the maximum impact on the risk of CP development in patients from different countries, as the products of these genes are key participants in the trypsin-dependent pathogenetic pathway of the pancreatitis development. Very often, patients with mutations in the *SPINK1* and *PRSS1* genes have a family history of CP in several generations, moreover, the age of the disease onset is usually much earlier compared to *CFTR*- or *CTCR*-associated CP [17]. Comparison of the current study results with similar studies conducted by foreign researchers revealed a rather large similarity in the spectrum of genetic findings in the *SPINK1* and *PRSS1* genes in the Russian and European patient populations. The most common pathogenic variants of the *SPINK1* gene in the Russian cohort include the missense variant c.101A>G (p.Asn34Ser, rs17107315) and the splicing site mutation c.194+2T>C (rs148954387); the recurrent pathogenic variant of *PRSS1* is c.86A>T (p.Asn29Ile, rs111033566), which, according to foreign researchers, occur in 5–21% of patients with this disease [4].

Specific variants of the *SPINK1* and *PRSS1* genes were also highlighted as requiring further study (*SPINK1*: rs370266754, rs199929811; *PRSS1*: rs569672741, rs182426777). For instance, the c.88-23A>T (rs199929811) variant of the *SPINK1* gene is annotated in the databases as a benign variant, but the results of the current study require revising clinical interpretation of this variant, as it was identified only in patients and was not found in anyone from the control group. A similar situation was seen for the rs569672741 and rs182426777 variants of the *PRSS1* gene.

Currently, the *CPA1* gene is the least studied among CP-predisposing genes. However, the results of the functional analysis of various changes in *CPA1* in the cell cultures and model animals indicate a significant contribution of some variants of this gene to the pathogenesis of the disease [22]. According to [4], *CPA1* gene variants in patients lead to an earlier onset of the disease. According to the results of our study, the following variants of the *CPA1* gene must be paid the greatest attention: c.586-30C>T (rs782335525), c.-90C>T (rs377553060), c.983A>G (p.Glu328Gly), and c.696+23_696+24delGG (chr7 130383816 TGG>TG,T). We are planning to conduct further study of these variants in the Russian cohort of patients with CP.

Therefore, the major contribution of the genetic causes of CP in Russian patients was observed (in descending order) for the *CTRC* gene variants (identified in 37.1% of cases), *CFTR* (18.1%), *SPINK1* (8.6%), *PRSS1* (8.6%), and *CPA1* (6.7%). This data is quite consistent with the results of the study in the European cohort of patients with CP [23]. At the same time, a large study in China revealed specific details that demonstrated the maximum impact of *SPINK1* (identified in 61.5%) and *PRSS1* (13.5%) gene variants on the risk of idiopathic pancreatitis development. The *CTRC* and *CFTR* genes variants were found in only 1.5 and 4.9% of cases, respectively [17]. This may prove the fact that the genetic diagnostics of CP should be performed taking into account the population characteristics. It is essential to pay attention to such characteristics during the development of genetic tests for genetic analysis of CP.

We could not find a genetic causative factors in 39% of CP patients, which is generally consistent with the data of foreign researchers [24]. Patients having no genetic risk factors identified during genetic testing might have additional genetic factors associated both with the polygenic nature of the pathology and with monogenic form of the disease, the clinical manifestations of which can include inflammation of the pancreatic tissue. For instance, it is known that some monogenic metabolic disorders (methylmalonic aciduria, impaired fatty acid oxidation, etc.) are characterized by clinical signs of CP in childhood and adolescence [25]. Meanwhile, identification of the exact genetic cause of the disease is of utmost importance.

Currently, the genetic characteristics of patients with CP allow to make a conclusion about the peculiarities of the course of the disease, its prognosis, and the probability of specific complications development, which primarily include pancreatic exocrine insufficiency, diabetes mellitus, and pancreatic cancer [26]. Taking into account the fact of active introduction of gene therapeutic technics and targeted therapy aimed to influence on the specific points of the molecular pathogenesis of the disease into clinical practice, it is obvious that the future will provide new opportunities for treatment of patients with CP based on their genetic profile. For instance, it was demonstrated that *CFTR* protein potentiators can reduce the number of pancreatitis attacks in patients with *CFTR*-associated pancreatitis [27].

## Conclusion

The developed genetic panel (*PRSS1*, *SPINK1*, *CTRC*, *CFTR*, and *CPA1* genes) for identification of genetic risk factors of the chronic pancreatitis development and the usage of the next-generation sequencing technology allowed to specify genetic predictors of the disease development in 61% of patients. Pathogenic and potentially pathogenic variants associated with the risk of this disease development were identified in the following genes: *CTRC* (37.1% of patients), *CFTR* (18.1%), *SPINK1* (8.6%), *PRSS1* (8.6%), and *CPA1* (6.7%). Recurrent variants of the *PRSS1*, *SPINK1*, *CTRC*, *CFTR*, and *CPA1* genes, contributing to the genetic predisposition to chronic pancreatitis development were identified in a cohort of Russian patients. It is assumed that the study results will be applied in genetic diagnostics and interpretation of the results of molecular genetic tests in chronic pancreatitis patients in the Russian Federation, as well as will help to increase the effectiveness of genetic analysis for families with chronic diseases of the gastrointestinal tract and, in particular, with diseases of the pancreas.
